
JOURNAL INFORMATION
==============================
NLM Title Abbreviation: Orthop Surg
Journal ID: Orthop Surg
NLM Journal ID: 101501666
Journal ID: OS

Orthopaedic Surgery

ISSN: 1757-7853
EISSN: 1757-7861
Publisher: Wiley

ARTICLE INFORMATION
==============================
PMCID: PMC11474667
DOI: 10.1111/os.13342
Article ID: OS13342
Article version: 1
Subjects: Issue Information, Issue Information

Issue Information

Electronic publication date: 2023 Aug 16
Publication date: 2023 Aug
Volume: 15
Issue: 8
Issue title: Shoulder and Elbow Surgery
First page: 1927
Last page: 1928
Copyright: © 2023 The Authors. Orthopaedic Surgery published by Chinese Orthopaedic Association and John Wiley & Sons Australia, Ltd.
License: This is an open access article under the terms of the http://creativecommons.org/licenses/by/4.0/ License, which permits use, distribution and reproduction in any medium, provided the original work is properly cited.
License URL: https://creativecommons.org/licenses/by/4.0/

Figure count: 0
Table count: 0
Page count: 2

source-schema-version-number: 2.0
cover-date: August 2023
details-of-publishers-convertor: Converter:WILEY_ML3GV2_TO_JATSPMC version:6.4.9 mode:remove_FC converted:15.10.2024

==============================

Volume 15 • Issue 8 • August 2023 • ISSN 1757-7861

### Orthopaedic Surgery

Open Access

SPECIAL ISSUE SHOULDER AND ELBOW SURGERY
GUEST EDITORS: MARIUS M. SCARLAT AND XIAO-MING WU

EDITORIAL

1929
SHOULDER AND ELBOW SURGERY SPECIAL ISSUE

REVIEW ARTICLES

1931
IS THERE ANY DIFFERENCE IN CLINICAL OUTCOME BETWEEN OPEN AND ARTHROSCOPIC TREATMENT FOR TENNIS ELBOW? A SYSTEMATIC REVIEW AND META-ANALYSIS
1944
COMPARISON OF EFFECTIVENESS AND SAFETY IN TREATING ACUTE ACROMIOCLAVICULAR JOINT DISLOCATION WITH FIVE DIFFERENT SURGICAL PROCEDURES: A SYSTEMATIC REVIEW AND NETWORK META-ANALYSIS

CLINICAL ARTICLES

1959
FRACTURE LINE MORPHOLOGY OF GREATER TUBEROSITY FRAGMENTS OF NEER THREE- AND FOUR-PART PROXIMAL HUMERUS FRACTURES
1967
THE CORRELATION BETWEEN VARIATION OF LABRAL ATTACHMENT AND LESIONS OF THE LONG HEAD OF THE BICEPS TENDON IN PATIENTS WITH ROTATOR CUFF TEARS
1975
THE EFFECT OF ARTHROSCOPIC EXTRA-ARTICULAR ENTIRE CORACOHUMERAL LIGAMENT RELEASE FOR PATIENTS WITH RECALCITRANT FROZEN SHOULDER
1983
OPEN ARTHROLYSIS FOR CHRONIC ELBOW DISLOCATION WITH EXTREMELY SEVERE STIFFNESS IN CHILDREN
1990
SHORT-TERM CLINICAL OUTCOME OF DUAL PLATE FIXATION IN THE TREATMENT OF PROXIMAL HUMERUS FRACTURES WITH CALCAR COMMINUTION
1997
THE CORRELATION BETWEEN VARIOUS SHOULDER ANATOMICAL INDICES ON X-RAY AND SUBACROMIAL IMPINGEMENT AND MORPHOLOGY OF ROTATOR CUFF TEARS
2007
INTRAMEDULLARY NAIL FOR TREATMENT OF PROXIMAL HUMERAL FRACTURE: A CREDIBLE FIXATION IN COMMINUTED CALCAR
2016
THE SAFETY ASSESSMENT OF IRRIGATION FLUID MANAGEMENT FOR SHOULDER ARTHROSCOPY AND ITS EFFECT ON POSTOPERATIVE EFFICACY
2025
A NOVEL AND OPEN CLASSIFICATION EMPHASIZING ON OSTEOLIGAMENTOUS COMPLEX FOR DISTAL CLAVICLE FRACTURES
2033
SURGICAL TREATMENT OF ADAMS TYPE IV ANTEROLATERAL FRACTURE OF THE ULNA CORONOID PROCESS
2042
MORPHOLOGICAL ANALYSIS OF FRACTURES OF THE PROXIMAL HUMERUS BY THE FRACTURE MAPPING TECHNIQUE
2052
THE CRITICAL SHOULDER ANGLE CAN BE ACCURATELY AND RELIABLY DETERMINED FROM THREE-DIMENSIONAL COMPUTED TOMOGRAPHY IMAGES
2062
COMBINED MEDIAL AND LATERAL APPROACH *VERSUS* PARATRICIPITAL APPROACH IN OPEN REDUCTION AND INTERNAL FIXATION FOR TYPE C DISTAL HUMERUS FRACTURE: A RANDOMIZED CONTROLLED STUDY
2074
EFFECT OF THE INITIAL DEFORMITY IN THE CORONAL PLANE ON POSTOPERATIVE OUTCOME OF PROXIMAL HUMERAL FRACTURES
2082
FUNCTIONAL AND MRI OUTCOMES AFTER IN SITU REPAIR VERSUS TEAR COMPLETION BEFORE REPAIR OF BURSAL-SIDE PARTIAL-THICKNESS ROTATOR CUFF TEARS
2091
CLINICAL AND RADIOGRAPHIC OUTCOMES OF TOTAL ELBOW ARTHROPLASTY USING A SEMI-CONSTRAINED PROSTHESIS WITH A TRICEPS-PRESERVING APPROACH OVER A MINIMUM FOLLOW-UP PERIOD OF 4 YEARS
2102
ELBOW KINEMATICS AND FUNCTION FOLLOWING TREATMENT WITH OPEN ARTHROLYSIS AND HINGED EXTERNAL FIXATOR
2110
COMPARISON OF CLINICAL OUTCOMES OF PLATELET-RICH PLASMA FOR EPICONDYLITIS, ELBOW: SIMULTANEOUS LATERAL AND MEDIAL VERSUS LATERAL VERSUS MEDIAL
2116
MID-TERM OUTCOMES OF ARTHROSCOPIC ROTATOR CUFF REPAIR IN PATIENTS WITH RHEUMATOID ARTHRITIS
2124
NOVEL CLAW-SHAPED BONE PLATE IN COMPLEX UNSTABLE SCAPULAR NECK AND BODY FRACTURES: COMPARISON WITH RECONSTRUCTION LOCKING PLATE
2132
A TRANSOSSEOUS SUTURE AS AN ALTERNATIVE TO SUTURE ANCHOR ON ANTERIOR-AVULSION GREATER TUBEROSITY FRAGMENT FIXATION IN NEER THREE-PART PROXIMAL HUMERAL FRACTURE: A BIOMECHANICAL STUDY

RESEARCH ARTICLES

2138
COMPARISON OF INTEROBSERVER AGREEMENT OF FOUR CLASSIFICATION SYSTEMS FOR LATERAL CLAVICLE FRACTURES BETWEEN TWO GROUPS OF SURGEONS: A MULTICENTER STUDY
2144
AN ANALYSIS OF THE MORPHOLOGY OF BONY BICIPITAL GROOVE FRACTURES IN PROXIMAL HUMERAL FRACTURES
2152
CANCELING NOTCH IMPROVES THE MECHANICAL SAFETY OF CLAVICLE LOCKING PLATE: A 3D FINITE ELEMENT STUDY

OPERATIVE TECHNIQUES

2157
ARTHROSCOPIC AUTOLOGOUS CORACOACROMIAL LIGAMENT AUGMENT TECHNIQUE FOR PARTICLE THICKNESS OF ROTATOR CUFF WITH AT LEAST TWO YEARS FOLLOW-UP
2167
A MODIFIED ARTHROSCOPIC OUTSIDE-IN SHOULDER RELEASE APPROACH FOR SEVERE SHOULDER STIFFNESS
2174
PARTIAL ARTICULAR SUPRASPINATUS TENDON AVULSION REPAIR AND PATCH: A TECHNICAL NOTE FOR AUGMENTING THE SUPRASPINATUS REINSERTION WITH THE LONG HEAD OF THE BICEPS TENDON

CASE REPORTS

2181
COMPLEX FUNCTIONAL POSTTRAUMATIC SHOULDER RECONSTRUCTION USING SHOULDER ARTHROPLASTY AND A PEDICLED INNERVATED LATISSIMUS DORSI FLAP—A CASE REPORT AND LITERATURE REVIEW
2187
CLINICAL APPLICATION OF NAIL-PLATE FIXATION IN THE TREATMENT OF COMPLEX PROXIMAL THIRD HUMERAL FRACTURE: A CASE REPORT AND LITERATURE REVIEW

WILEY

CHINESE ORTHOPAEDIC ASSOCIATION
中华医学会骨科学分会

**View this journal online at wileyonlinelibrary.com/journal/os**

### Orthopaedic Surgery

**Editor-in-Chief**
Gui-xing Qiu, *Beijing, China*
Francis John Hornicek, *Boston, USA*

**Associate Editor**
Yong-cheng Hu, *Tianjin, China*
Thomas Blattert, *Leipzig, Germany*
Douglas Letson, *Tampa, USA*
Xin-long Ma, *Tianjin, China*
Ling Qin, *Hong Kong, China*

**Editorial Board**
Giovanni Alessi, *Gent, Belgium*
Haluk Berk, *Izmir, Turkey*
Thomas Blattert, *Leipzig, Germany*
Mathis Bostrom, *New York, USA*
Kao-wha Chang, *Taiwan, China*
Edmund YS Chao, *Baltimore, USA*
Liao-bin Chen, *Wuhan, China*
Yan-xi Chen, *Shanghai, China*
Kenneth MC Cheung, *Hong Kong, China*
Thomas L. Clemens, *Birmingham, USA*
Benny Thellefsen Dahl, *Copenhagen, Denmark*
Lian-fu Deng, *Shanghai, China*
Jean Destandau, *Bordeaux, France*
Jian Dong, *Shanghai, China*
Shun-wu Fan, *Hangzhou, China*
David Fang, *Hong Kong, China*
Shi-jun Gao, *Shijiazhuang, China*
Wei Guo, *Beijing, China*
Yong Hai, *Beijing, China*
Alan R. Hargens, *San Diego, USA*
Francis John Hornicek, *Boston, USA*
William C. Horton, *Atlanta, USA*
John Hsiang, *Seattle, USA*
Yong-cheng Hu, *Tianjin, China*
Qing Jiang, *Nanjing, China*
Paul Jutte, *Groningen, Netherlands*
Jean Pierre Kalala, *Gent, Belgium*
Choon-Ki Lee, *Seoul, Korea*
Jean Jacque Lehuec, *Bordeaux, France*
Lawrence G. Lenke, *Seattle, USA*
Douglas Letson, *Tampa, USA*
Kwok Sui Leung, *Hong Kong, China*
Jian-hao Lin, *Beijing, China*
Bao-ge Liu, *Beijing, China*
Wei-hee Lo, *Taiwan, China*
William Weijia Lu, *Hong Kong, China*
Keith DK Luk, *Hong Kong, China*
Zong-ping Luo, *Suzhou, China*
Cyril Mauffrey, *Denver, USA*
Wei-hu Ma, *Ningbo, China*
Xin-long Ma, *Tianjin, China*
Ling Qin, *Hong Kong, China*
Gui-xing Qiu, *Beijing, China*
Axel Reinhardt, *Potsdam, Germany*
Pu-yi Sheng, *Guangzhou, China*
Harry L Shufflebarger, *Miami, USA*
David Stulberg, *Chicago, USA*
Paul Sung, *San Diego, USA*
Paul Unwin, *Leicester, UK*
Luc F. De Waele, *Gent, Belgium*
Fei Wang, *Shijiazhuang, China*
Wei-jun Wang, *Nanjing, China*
James N. Weinstein, *Chicago, USA*
Hee-Kit Wong, *Singapore*
Su-jia Wu, *Nanjing, China*
Jian-ru Xiao, *Shanghai, China*
Hui-lin Yang, *Suzhou, China*
Tzai-chiu Yu, *Taiwan, China*
Xi-jie Yu, *Chengdu, China*
Hansen A. Yuan, *New York, USA*
Hong-wen Zhu, *New York, USA*

---

**Address for Editorial Correspondence:** Editorial Board of *Orthopaedic Surgery*, Tianjin Hospital, 122 Munan Road, Tianjin 300050, China (email: orthopaedicsurgery@126.com; telephone: +86 22 2833 4734; fax: +86 22 2824 1184).

**Disclaimer:** The Publisher, Tianjin Hospital and Editors cannot be held responsible for errors or any consequences arising from the use of information contained in this journal; the views and opinions expressed do not necessarily reflect those of the Publisher, Tianjin Hospital and Editors, neither does the publication of advertisements constitute any endorsement by the Publisher, Tianjin Hospital and Editors of the products advertised.

**Copyright © 2023 The Authors. Orthopaedic Surgery published by Tianjin Hospital and John Wiley & Sons Australia, Ltd**

**Copyright and Copying (in any format)**
Copyright © 2023 The Authors. Orthopaedic Surgery published by Tianjin Hospital and John Wiley & Sons Australia, Ltd. All rights reserved. No part of this publication may be reproduced, stored or transmitted in any form or by any means without the prior permission in writing from the copyright holder. Authorization to copy items for internal and personal use is granted by the copyright holder for libraries and other users registered with their local Reproduction Rights Organisation (RRO), e.g. Copyright Clearance Center (CCC), 222 Rosewood Drive, Danvers, MA 01923, USA (www.copyright.com), provided the appropriate fee is paid directly to the RRO. This consent does not extend to other kinds of copying such as copying for general distribution, for advertising or promotional purposes, for republication, for creating new collective works or for resale. Permissions for such reuse can be obtained using the RightsLink "Request Permissions" link on Wiley Online Library. Special requests should be addressed to: permissions@wiley.com

For submission instructions, subscription and all other information visit: http://wileyonlinelibrary.com/journal/os

ISSN 1757-7861 (Online)

OS.JEB.August23
